# Retention period differentially attenuates win–shift/lose–stay relative to win–stay/lose–shift performance in the rat

**DOI:** 10.3758/s13420-017-0289-7

**Published:** 2017-09-22

**Authors:** Phil Reed

**Affiliations:** Department of Psychology, Swansea University, Singleton Park, Swansea, SA2 8PP UK

**Keywords:** Win–shift, Win–stay, Lose–stay, Lose–shift, Retention interval

## Abstract

Hungry rats were trained in a two-lever conditioning chamber to earn food reinforcement according to either a win–shift/lose–stay or a win–stay/lose–shift contingency. Performance on the two contingencies was similar when there was little delay between the initial, information part of the trial (i.e., win or lose) and the choice portion of the trial (i.e., stay or shift with respect to the lever presented in the information stage). However, when a delay between the information and choice portions of the trial was introduced, subjects experiencing the win–shift/lose–stay contingency performed worse than subjects experiencing the alternative contingency. In particular, the lose–stay rule was differentially negatively impacted relative to the other rules. This result is difficult for ecological or response interference accounts to explain.

Many species tend to show better performance on a spatial task that requires them to shift a response from one that has just been reinforced on the preceding trial to a different response on the next trial (win–shift) than when they have to maintain the previously reinforced response (win–stay). Such a win–shift performance superiority has been observed in, among other species, rats (Cohen, Westlake, & Szelest, 2004; Olton & Samuelson, 1976), nectar-feeding birds (Burke & Fulham, 2003), pigs (Laughlin & Mendl, 2000), and echidnas (Burke, Cieplucha, Cass, Russell, & Fry, 2002). However, this win–shift performance superiority has not always been noted, and it can be elusive in the conditioning chamber (cf. Evenden & Robbins, 1984; Morgan, 1974; Randall & Zentall, 1997; Reed, 2016). In fact, the emergence of the win–shift superiority is dependent on the precise nature of the contingencies in operation. One factor that has been suggested as being important in determining the emergence of win–shift performance superiority is the gap between the initial and subsequent responses (the interresponse interval; IRI). In fact, stronger win–shift than win–stay behavior is typically most pronounced when the IRI is short in duration, whereas win–stay performance can become superior at longer IRIs (Burke & Fulham, 2004).

This tendency toward superior win–shift as compared to win–stay performance has been linked to ecological adaptations to environments that make it optimal for individuals not to return to a depleted feeding patch after a foraging bout (see Krebs & McCleery, 1984; Rayburn-Reeves, Stagner, Kirk, & Zentall, 2013; Timberlake, 2001), or to a patch that may have become aversive due to a buildup of protective chemicals unleashed by the prey (Burke et al., 2002). The change from win–shift to win–stay strategies with an increasing IRI has also been suggested to reflect optimal foraging principles (see Burke & Fulham, 2003; Timberlake, 2001). As time passes from the original patch visit, the likelihood of food being at that patch increases, and win–stay behavior might then be the optimal foraging strategy (Burke & Fulham, 2003).

Although this finding of a change from win–shift to win–stay behavior with a longer IRI is theoretically intriguing, it should be noted that the finding has not been universally replicated (see Burke et al., 2002; Randall & Zentall, 1997; Shimp, 1976). The studies that have failed to report this finding have typically not employed species that forage in patch-depleting environments, such as echidnas (Burke et al., 2002) and pigeons (Randall & Zentall, 1997; Shimp, 1976). Given the foraging ecology of these species, it might be argued that these failures to observe the change from shift to stay behavior with longer IRIs might also be predicted by evolutionary constraints.

However, it has been noted that these win–shift effects are also sometimes not seen in rats, especially when this species is tested in a conditioning chamber, rather than using maze apparatus (e.g., Morgan, 1974; Reed, 2016). For example, Reed (2016) noted a deficit in win–shift, as compared to win–stay, performance (and also as compared to lose–stay and lose–shift performance). In this study, hungry rats learned to lever-press according to win–stay, win–shift, lose–stay, and lose–shift rules. During this training, a lever (left or right) would be inserted into a conditioning chamber, and a response to that lever would or would not be reinforced. Following this part of the trial, both levers would be inserted, and the rat would have to press a lever according to a stay or shift rule, depending on whether or not the previous response to the single lever had been reinforced (i.e., under a win–shift contingency, the rat would have to press the opposite lever from the one that was just reinforced, but under a win–stay rule would have to press the same lever). In a first experiment, which compared acquisition performance on win–stay/lose–shift rules versus win–shift/lose–stay, the two lose rules were learned more quickly than the two win rules, irrespective of the contingency. In Experiment 2, when the rules were taught separately, the lose rules and the win–stay rule were all learned faster than the win–shift rule.

In fact, this finding reported for rats by Reed (2016) was also broadly similar to that noted by Randall and Zentall (1997) using a conditioning-chamber procedure for pigeons. Given that some studies have suggested that rats, on whom much of the data relating to optimal foraging as an explanation of win–shift performance is based, do not always show predicted patterns of behavior, the present study was initially intended to establish the impact of increasing an IRI on win–shift/lose–stay and win–stay/lose–shift behavior for this species in the conditioning chamber. If optimal-foraging views are correct, then it might be expected that there would be a change from better win–shift/lose–stay performance to better win–stay/lose–shift performance with an increased IRI.

The ecologically oriented view above is not the only suggestion as to why these effects are noted. In fact, Randall and Zentall (1997; see also Reed, 2016) reported little difference between win–stay/lose–shift and win–shift/lose–stay performance at short IRIs in the conditioning chamber for pigeons, but noted an increase in the win–stay/lose–shift tendency as the IRI increased. Randall and Zentall suggested that the act of eating (which involves moving away from the response manipulandum in the chamber) after the first response on win trials interferes with the ability to remember the position of the response on that trial. This would make subsequent shift or stay behavior harder to master on win than on lose trials, in which there is no such interference. With short IRIs, it was argued that this would make little difference to overall performance, but as the IRI increases, Randall and Zentall predicted that lose trials should be performed better than win trials (which has been noted; see Randall & Zentall, 1997, Exp. 1; Reed, 2016, Exp. 1). However, when the differential effect of such interfering eating responses was eliminated (Randall & Zentall, 1997, Exp. 2), the results suggested that rules, apart from the win–stay rule that is based on reinforcement learning, would be equally badly affected by an increased IRI—making the win–stay/lose–shift performance overall better than win–shift/lose–stay performance. Although Reed also noted a lose performance superiority, as compared to win, for rats in a conditioning chamber with a minimal IRI (1 s), it has not been discovered whether increasing the IRI would have the same impact for rat subjects on a similar task.

However, one feature of the Randall and Zentall (1997) data that was not commented upon suggests that interfering responses may not be the only factor mediating these effects. In Experiment 2 of the Randall and Zentall report, when the effect of such interfering responses was minimized, performance according to the various individual rules (e.g., win–stay, lose–shift, etc.) showed a differential decrease in accuracy as a result of increasing the IRI from 0 to 8 s. Appropriate behavior at the longer IRI was performed at over 75% of the 0-s IRI accuracy for the win–shift (68% at 0s and 56% at 8s), win–stay (88% to 66%), and lose–shift (70% to 56%) rules. In contrast, the lose–stay rule was performed at only about 60% accuracy (80% to 50%). This differential effect could not be predicted on the basis of any straightforward version of the ecological view. Neither could it be explained on the basis of interfering responses, since both lose rules should have been equally impacted. These data suggest that further exploration of the impact of increasing the IRI on performance on win–stay/lose–shift and win–shift/lose–stay performance might well provide data challenging to existing theory.

Given these considerations, the present study used a win–shift/lose–stay and win–stay/lose–shift procedure in the conditioning chamber for rats. The rats were presented with an initial trial (i.e., they responded to a particular lever) that either did or did not result in reinforcement, and then they were presented with both levers after an IRI and received reinforcement for pressing one of the levers, depending on the reinforcement rule in operation (i.e., for pressing the same lever that was just reinforced in a win–stay trial, etc.). The aim was to see whether there was any advantage in performance for one contingency over the other, as there is for win–shift in a maze apparatus (Cohen et al., 2004; Olton & Samuelson, 1976), but not in the conditioning chamber (Morgan, 1974; Reed, 2016). In addition, the study also aimed to determine whether win–stay/lose–shift performance would come to be stronger than win–shift/lose–stay performance as the IRI increased, as has been found previously in several settings (Burke & Fulham, 2003; Randall & Zentall, 1997). Finally, the impact on each of the four rules separately was examined, to explore whether the numerical effect seen in the data reported by Randall and Zentall for pigeons could be confirmed with rats. The latter finding could provide challenges for several existing views.

## Method

### Participants

Twenty-four male, Long Evans hooded rats served in the present experiment. The rats were 8–9 months old and had a free-feeding body weight range of 320–490 g at the start of the experiment. They were maintained at approximately 80% of this weight throughout the study. The subjects had extensive experience of either the win–stay/lose–shift or win–shift/lose–stay contingencies to be used in the present experiment (see Reed, 2016, Exp. 1). The rats were housed individually and had constant access to water in their home cages.

### Apparatus

Eight identical operant conditioning chambers (Campden Instruments Ltd.) were used. Each chamber was housed in a light- and sound-attenuating case. A 65-dB(A) background masking noise was supplied by a ventilating fan. Each chamber was equipped with two retractable levers. The food tray, into which reinforcement (one 45-mg food pellet) could be delivered, was covered by a clear, hinged Perspex flap and was centrally located between the two levers. A light could be operated to illuminate the magazine tray on delivery of reinforcement. A houselight was located centrally on the same chamber wall as the magazine tray and response levers.

### Procedure

Since the subjects had previously been exposed to win–shift/lose–stay or win–stay/lose–shift contingencies, they needed no magazine or leverpress training. For the present experiment, the two groups (win–stay/lose–shift and win–shift/lose–stay) were treated identically, except for the rule that governed the delivery of reinforcement. During the intertrial interval (ITI), which lasted 30 s, the houselight was off and both levers were retracted from the chamber. Each trial consisted of two elements: an information stage and a choice stage. The houselight was illuminated throughout each trial.

For the information stage, one lever was randomly selected, on a 50:50 basis, and presented for 15 s. If the rat did not fulfill the response requirement of three responses, the lever was withdrawn and an ITI of 30 s commenced. If the rat completed the response requirement within the specified time, the lever was withdrawn, and the trial continued. In addition to the lever withdrawal, completion of the response requirement sometimes led to the delivery of a food pellet (i.e., on a win trial) and sometimes did not (i.e., a lose trial). On win trials, the tray light was illuminated for a 1-s interresponse interval (IRI), after which both levers were inserted into the chamber for the choice stage. On the lose trials, no food pellet was delivered and the food tray was not illuminated, but, after a 1-s IRI, both levers were again presented to the subject for the choice stage.

The identity of the correct lever during the choice stage was determined by a combination of the identity of the lever in the information stage and the outcome of the response. During the choice stage, rats with the win–shift/lose–stay contingency were required to press the lever that had not been presented in the information stage, if reward had been given, but were required to press the lever that had been presented in the information stage, if no reward had been presented. Rats with the win–stay/lose–shift contingency were required to press the same lever that had been presented in the information stage, if reward had been given, but to press the lever that had not been presented in the information stage, if no reward had been given during that stage. The choice stage was complete when the rat had made the required number of leverpresses (three) on one of the levers within 15 s of the trial commencing. Both levers were then withdrawn. If the response requirement had been correctly made, then a food pellet was delivered and the tray light was illuminated for 5 s, after which the ITI began. If the responses had been emitted to the lever not specified in the rule, then no food pellet was delivered and there was a 5-s period of darkness prior to the ITI commencing. If the rat failed to complete the requirement on one lever within 15 s, the levers were withdrawn, the trial was abandoned, and the ITI commenced.

Sessions lasted until the rat had completed 40 trials or until 40 min had elapsed. A maximum of 20 trials of each type (win or lose) were completed in a session. The nature of the trial (win or lose) was determined randomly, with the exceptions that no more than three trials of a particular type could occur in a row, and when 20 trials of a particular type had been delivered, the remaining trials were of the other type.

In a previous study (Reed, 2016, Exp. 1), the subjects had been exposed to the above contingencies for 33 sessions. For the first two sessions of the present experiment, the IRI for all rats remained at 1 s, as it had been during the previous 33 sessions of training. The IRI was then increased to 5 s for the next two sessions, and then returned to 1 s for Sessions 5 and 6. For Sessions 7 and 8 the IRI was again increased to 5 s, after which it was reduced to 1 s for the final two sessions of training. Thus, the study had a mixed-model design, with Group (win–stay/lose–shift versus win–shift/lose–stay) as a between-subjects factor and IRI (1 vs. 5 s) as a within-subjects factor.

## Results

The number of trials in which the rats received reinforcement (i.e., performed correctly according to the rule) on each contingency during a session was calculated as a percentage of the total number of exposures to trials with that contingency the rat had received during that session. The overall acquisition of the two contingencies (i.e., the percentages of trials correct) followed courses largely similar to those noted prior to the present experimental manipulations—with the lose rules being learned more quickly than the win rules (see Reed, 2016, Exp. 1). After 33 sessions of either win–shift/lose–stay or win–stay/lose–shift contingencies, the rats trained on the latter contingencies had reached a group-mean performance level at which 79.7% of the trials were performed correctly, as compared to a score of 85.9% of the trials performed correctly by the win–shift/lose–stay group.

A mixed-model analysis of variance (ANOVA) conducted for the acquisition of these two contingencies in the 33 sessions of training, with Group (win–shift/lose–stay vs. win–stay/lose–shift) as a between-subjects factor and Session as a within-subjects factor, revealed a significant main effect of session, *F*(32, 704) = 32.78, *p* < .001, *η*_p_^2^ = .598, indicating an improvement over training. Cohen (1988) suggested that a *η*_p_^2^ = _*.*_01 be regarded as a small effect size, *η*_p_^2^ = *.*06 as a medium effect size, and *η*_p_^2^ = _*.*_14 as large. No significant main effect of group emerged, nor an interaction between the factors: both *F*s < 1, largest *η*_p_^2^ = .008.

**Fig. 1:**
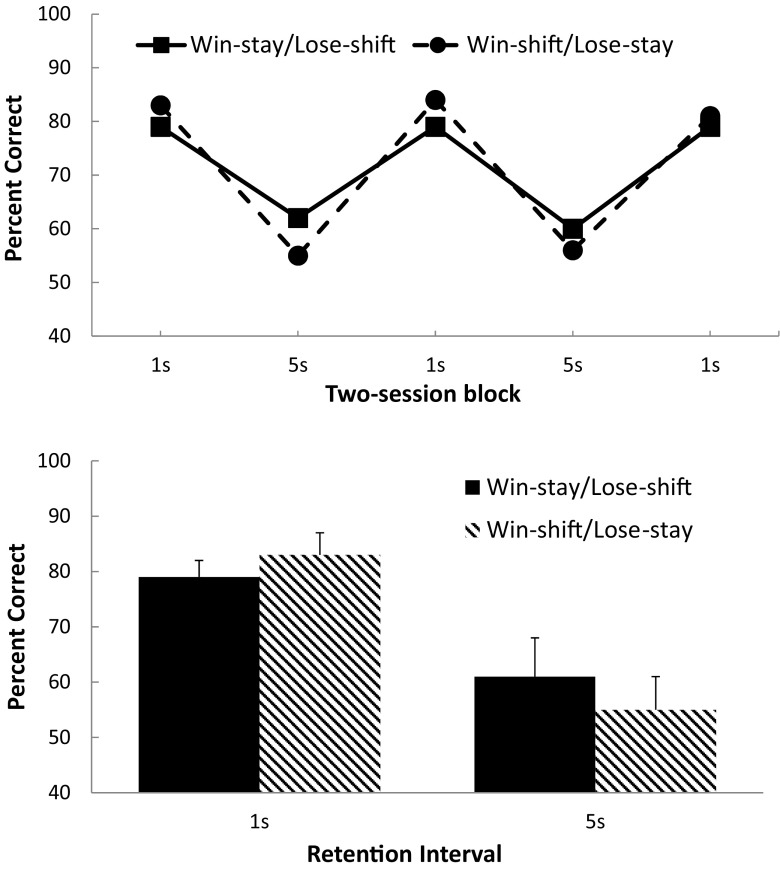
(Top-panel) Group-mean percentage accuracy for the win–stay/lose–shift and win–shift/lose–stay contingencies at each replication of each interresponse time. (Bottom panel) Group-mean percentage accuracy for the win–stay/lose–shift and win–shift/lose–stay contingencies at each interresponse time.

The top panel of Fig. 1 displays the group-mean percentages of trials correct for both groups (collapsed over the two rules for each contingency) for the three two-session blocks of 1-s IRI testing and for the two two-session blocks of 5-s IRI testing. It can be observed that on the initial and subsequent phases in which the IRI was 1 s, the win–shift/lose–stay group performed slightly more accurately than the win–stay/lose–shift group. This pattern of results was generally observed in all three replications of the 1-s IRI and indicates a recoverable performance (although, on the last replication of the 1-s IRI, this win–shift/lose–stay performance superiority was somewhat smaller than on the previous replications). In contrast, with a 5-s IRI, the behavior specified by the win–stay/lose–shift contingency was performed at a higher level of accuracy than that specified by the win–shift/lose–stay contingency. This pattern of results was observed for both replications of the 5-s IRI, but again, it should be noted that this difference was more pronounced on the first replication than on the second.

The bottom panel of Fig. 1 shows the group-mean performance (i.e., percentages of trials correct) collapsed across the three blocks of 1-s and 5-s IRI trials, separately, and shows the switch to superior win–stay/lose–shift performance at the longer IRI. A two-factor mixed-model ANOVA (Group × Retention Interval) was conducted on the data shown in the bottom panel of Fig. 1, and this revealed no main effect of group, *F* < 1, *η*_p_^2^ = .007, but a significant main effect of retention interval, *F*(1, 22) = 103.37, *p* < .001, *η*_p_^2^ = .825, and a significant interaction between the factors, *F*(4, 88) = 4.11, *p* = .05, *η*_p_^2^ = .157. To explore the interaction further, simple-effect analyses were conducted comparing the contingencies at each retention interval. There was no significant difference between the contingencies at the 1-s IRI, *F*(1, 22) = 1.01, *p* > .80, *η*_p_^2^ = .049, but there was a significant difference at the 5-s IRI, *F*(1, 22) = 4.64, *p* = .05, *η*_p_^2^ = .140.

**Fig. 2:**
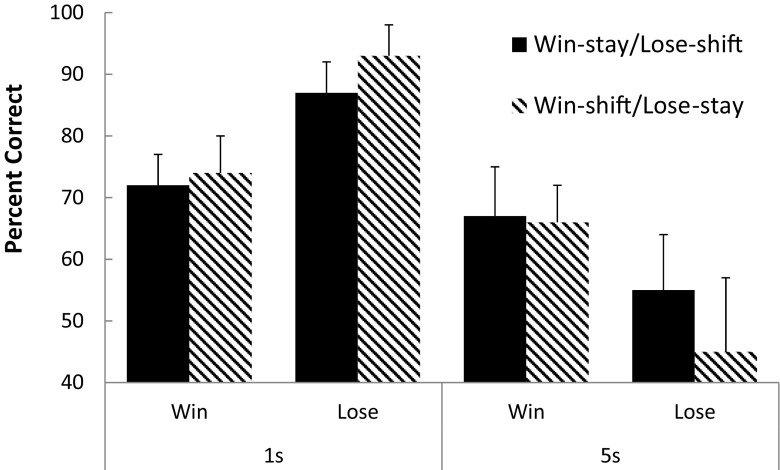
Group-mean percentage accuracy for each rule of the win–stay/lose–shift and win–shift/lose–stay contingencies at the 1- and 5-s interresponse times.

Figure 2 shows the group-mean percentage of trials correct for each rule (collapsed across the repetitions of 1-s and 5-s IRIs) for each group (i.e., win–stay/lose–shift and win–shift/lose–stay). Inspection of these data reveals that, for the 1-s IRI, the behavior engendered by the lose rule was performed slightly more accurately than that for the win rule for both the win–stay/lose–shift and win–shift/lose–stay groups. The accuracy of performance for both rules in both groups dropped with the introduction of the 5-s IRI. However, the increased IRI impacted performance more on the lose rules than on the win rules. This reduction in lose-rule performance was especially pronounced for the lose–stay rule.

A three-factor mixed-model ANOVA (Group × Rule × Interval) was conducted on these data and revealed no significant main effect of group, *F* < 1, *η*_p_^2^ = .007, or rule, *F* < 1, *η*_p_^2^ = .001, but a significant main effect of interval, *F*(1, 22) = 103.37, *p* < .001, *η*_p_^2^ = .825. No significant interaction emerged between group and rule, *F* < 1, *η*_p_^2^ = .001, but there were significant interactions between group and interval, *F*(1, 22) = 4.11, *p* = .05, *η*_p_^2^ = .157, and rule and interval, *F*(1, 22) = 110.65, *p* < .001, *η*_p_^2^ = .834, and between all three factors, *F*(1, 22) = 4.69, *p* = .05, *η*_p_^2^ = .159.

To further analyze the three-way interaction, separate two-factor ANOVAS (Group × Interval) were conducted on the win and lose rules (Howell, 1997). The ANOVA conducted on the win rule revealed a significant main effect of interval, *F*(1, 22) = 11.76, *p* = .002, *η*_p_^2^ = .348, but no significant main effect of group, *F* < 1, *η*_p_^2^ = .001, or interaction between group and interval, *F* < 1, *η*_p_^2^ = .029. The ANOVA conducted on the lose rule revealed no significant main effect of group, *F* < 1, *η*_p_^2^ = .011, but a significant main effect of interval, *F*(1, 22) = 128.85, *p* < .001, *η*_p_^2^ = .854, and a significant Group × Rule interaction, *F*(1, 22) = 4.83, *p* = .03, *η*_p_^2^ = .181. To analyze the interaction, simple effect analyses were conducted on the lose-rule performance between the groups at each retention interval, which revealed no significant difference at the 1-s interval, *F* < 1, *η*_p_^2^ = .002, but a significant group difference for the lose rule at the 5-s IRI, *F*(1, 22) = 4.49, *p* = .05, *η*_p_^2^ = .149.

## Discussion

The present study was conducted to explore the impact of an IRI on win–stay/lose–shift and win–shift/lose–stay performance for rats in a conditioning chamber. Previous studies with other species had suggested that increasing the IRI should lead to an increase in the accuracy of win–stay/lose–shift performance (Burke & Fulham, 2003). The present results suggested little difference in the levels of win–shift/lose–stay and win–stay/lose–shift performance with a 1-s IRI—with the former contingency being performed slightly more accurately than the latter (at least, on the first two replications conducted here). However, win–stay/lose–shift behavior was performed more accurately when the IRI was increased to 5 s.

This finding replicates many previous studies that have explored this phenomenon for species that forage in patch-depleting areas. To this extent, the data, as they relate to the impact of an increasing IRI, are generally consistent with what might be predicted on the basis of ecological views of optimal foraging (e.g., Krebs & McCleery, 1984; Timberlake, 2001)—although it should be noted that the lack of a win–shift/lose–stay performance superiority at a shorter IRI (see also Morgan, 1974; Randall & Zentall, 1979; Reed, 2016) is not entirely consistent with this view.

An issue that should be noted in this regard that there were differences between the levels of familiarity with the two retention intervals in this study; the rats had been trained on the 1-s IRI, and were then transferred to the 5-s IRI. This introduces the possibility that the present results were due to unfamiliarity with the 5-s IRI. That the effect of the 5-s IRI did appear to reduce marginally in magnitude over repetitions would be consistent with this view. In fact, this procedural issue is true for almost all studies of the impact of an IRI on win–stay versus win–shift type performance (see also Randall & Zentall, 1977). This is perhaps not a surprising way of training and testing animals, because it may well take a substantial period to train animals initially on a 5-s IRI, making cross-group comparisons with a group trained for an equivalent period on a 1-s period difficult. However, a problem with suggesting that the effect of the IRI might be one of familiarity is that it appeared to differentially impact one contingency (win–stay/lose–shift) to a greater extent than the other. Thus, even if the effect was one of unfamiliarity with the IRI aspect of the contingency, it should be acknowledged that this unfamiliarity disrupted performance more on the win–shift/lose–stay contingency than on the win–stay/lose–shift rule. Most ecological accounts of such performance claim that performance involving a win–shift rule is learned more readily, and as such, it might be expected to withstand disruption more strongly than behavior based on a win–stay contingency, whatever the source of that disruption—memorial or unfamiliarity (Nevin & Grace, 2000).

However, a second aim of the study was determine whether the increased IRI would differentially impact performance on the individual rules of the two contingencies (i.e., win–stay, win–shift, lose–stay, and lose–shift). This was premised on a reanalysis of the data on pigeons provided by Randall and Zentall (1997), which had suggested that accuracy on the lose–stay rule was impacted worst by the increased IRI. The present study with rats confirmed this observation. This finding is not entirely consistent with many views of win–shift versus win–stay behavior. An ecological view (e.g., Burke & Fulham, 2003) would suggest that both win–shift and lose–stay behavior might be negatively impacted by an increase in the IRI; as time passes from an original patch visit, the likelihood of food being found there again increases, promoting win–stay behavior. In contrast, an interfering-response view (Randall & Zentall, 1997) would suggest that both win rules should be affected worse than the lose rules by such an increase. Moreover, a speculative view suggested by Reed (2016) of why lose rules appear to be learned more readily than win rules fares equally badly with these data. This view suggests that the value of the reinforcer (its power to maintain behavior) will be a function of the amount of “effort” (e.g., the number of responses) expended in order to obtained that reward (e.g., Lea, 1981; Rachlin, Battalio, Kagel, & Green, 1981). During lose trials, the overall amount of reinforcement presented to the subjects is less than on win trials (i.e., a total of one instead of two reinforcers). This may tend to increase the value of the reinforcer presented on those trials, due to the greater number of responses needed to secure it, which, in turn, promotes learning of the lose contingencies. However, if that were true, behavioral-momentum theory would suggest that this learning should be more, not less, resistant to disruption (Nevin & Grace, 2000), which it is not.

However, a learning-theory-based view might explain this pattern of data. It may be that experiencing a lose trial results in an aversive, frustrative state (Amsel, 1992), which produces a short-term tendency to avoid the recently presented lever (see Leitenberg, 1965, for a discussion in a procedure related to the present one). If it is assumed that such aversion increases with time out from reward (Leitenberg, 1965), then this aversive state would be strongest in lose trials after the longer IRI. A typical response of an animal to an aversive state is avoidance, so this would tend to promote lose–shift behavior but would work against lose–stay behavior. This account is clearly speculative and needs further testing. One immediate problem with such a view is that, if the absence of reinforcement on lose trials causes conditioned avoidance of the lever associated with the absence of reinforcement, it is unclear why this only happens with a 5-s IRI. It may be, of course, that the 5-s IRI strengthens the conditioned avoidance, making the effect especially pronounced on these trials, but there is no avoidance at all with the 1-s IRI, and in fact, accuracy was highest for this combination of contingencies. Therefore, it remains to be explained why avoidance only develops with a longer IRI.

Other possibilities exist regarding why there might be a differential pattern of impact of the IRI. It might be that nonreward establishes a memory that weakens quickly over 5 s, although this would not account for the differential effect of the IRI on lose–stay performance. Another consideration might be that rats are near the feeder when the levers are extended on all 1-s trials, and stay near the feeder longer consuming the pellet on win trials. On lose trials with a 5-s delay, however, they may move away from the lever (more readily than they do on 1-s trials) and subsequently choose the nearest lever when the levers are reextended in the choice phase. However, again this does not explain the differential lose–stay versus lose–shift accuracy difference.

The reasons why the typical win–shift performance superiority that is seen in the context of a maze study is not seen in a conditioning chamber is currently unclear. Of course, the present conditioning-chamber procedure is quite different from the maze studies that form the basis for many theories of such performance. For example, rats encounter food in different arms of the maze and not in a single feeder, they enter the arm to find the food and do not press a lever to have it delivered, and the delay between entering an arm and a subsequent opportunity to reenter the arm is substantially longer than 5 s. It may be that any of these procedural differences are responsible for the discrepant findings. The spatial aspects of the task are salient in a maze and may favor a win–shift strategy (Olton & Samuelson, 1976). In a conditioning chamber, but not in a maze, reinforcement is obtained from the same location (the food magazine), regardless of the response made on the initial portion of the trials. Alternatively, what happened on the last trial may be more salient in the conditioning chamber than in the maze for rats (Rayburn-Reeves et al., 2013). However, that the data from this study and others (Randall & Zentall, 1997; Reed, 2016) replicate some aspects of the findings from maze studies suggests that common mechanisms may be at play. Again, further work will be needed to parse these possibilities.

In summary, the present report has shown that win–stay/lose–shift behavior is performed more accurately than win–shift/lose–stay behavior with a longer IRI. This has been found for other species in a variety of apparatuses. However, the present report suggested that the lose–stay rule is the one most affected by this change, which presents some difficulties for many contemporary theories to explain.
